# Retrospective clinical outcomes in the definitive treatment of high-energy tibial diaphyseal fractures using hexapod external fixator versus monolateral external fixator

**DOI:** 10.1186/s12891-022-05257-1

**Published:** 2022-04-08

**Authors:** Yanshi Liu, Kai Liu, Feiyu Cai, Xingpeng Zhang, Hong Li, Tao Zhang, Chuang Ma, Aihemaitijiang Yusufu

**Affiliations:** 1 Department of Trauma and Microreconstructive Surgery, the First Affiliated Hospital of Xinjiang Medical University, Urumqi, Xinjiang China; 2 Department of Orthopedics, Shanghai Pudong New Area People’s Hospital, Shanghai, China; 3 Department of Orthopedics, Zigong Fourth People’s Hospital, Zigong, Sichuan China; 4 Department of Orthopedics and Trauma, Tianjin Hospital, Tianjin, China

**Keywords:** External fixation, Hexapod external fixator, High-energy trauma, Monolateral external fixator, Tibial diaphyseal fracture

## Abstract

**Background:**

External fixation, which can preserve the biomechanical microenvironment of fracture healing, plays an important role in managing the high-energy fractures with poor surrounding soft tissues. The purpose of this study was to determine the differences of clinical outcomes, if any, between hexapod external fixator and monolateral external fixator in the definitive treatment of high-energy tibial diaphyseal fractures.

**Methods:**

A total of 53 patients with high-energy tibial diaphyseal fractures and definitively treated by the hexapod external fixator (HEF) or monolateral external fixator (MEF) were retrospectively collected and analyzed, from March 2015 to June 2019. There were 31 patients in the HEF treatment, and the other 22 patients were managed by the MEF. The demographic data, surgical duration, external fixation time, final radiological results, complications, and clinical outcomes were documented and analyzed. Difficulties that occurred during the treatment were classified according to Paley. The clinical outcomes were evaluated by the Association for the Study and Application of the Method of Ilizarov criteria (ASAMI) at the last clinical visit.

**Results:**

The mean surgical duration in the HEF group (62.4 ± 8.3 min) was shorter than that in the MEF group (91.4 ± 6.9 min) (*P* < 0.05). All patients acquired complete bone union finally. Patients in the HEF group (24.2 ± 3.1 weeks) underwent a shorter average external fixation time than that in the MEF group (26.3 ± 3.8 weeks) (*P* < 0.05). Satisfactory alignment was achieved in all patients without the need for remanipulation. The residual sagittal plane deformities in the HEF group were all less than that in the MEF group (*P* < 0.05). The complication rate was 35.5% in the HEF group, while 45.5% in the MEF group. There was no statistically significant difference between the two groups in ASAMI scores (*P* > 0.05).

**Conclusion:**

There is no statistically significant difference in finally clinical outcomes between hexapod external fixator and monolateral external fixator in the definitive treatment of high-energy tibial diaphyseal fractures. The hexapod external fixation treatment is a superior effective method, including advantages of stable fixation, less surgical duration, postoperatively satisfactory fracture reduction, and fewer complications.

## Background

The optimal definitive treatment of high-energy complex fractures remains a challenging problem in the orthopedic scenario. Current alternative treating options include intramedullary nailing, plate fixation, external fixation, or combining these methods [1, 2]. Although open reduction and internal fixation (ORIF) which contributes to anatomic reduction has acted as the gold standard for diaphyseal fractures treatment, it is the most invasive form with potential infection risk, especially for high-energy fractures with severe soft tissue damage. Meticulous soft tissue care combined with external fixation has given satisfactory clinical results in recent years [3–9].

External fixators have played a crucial and effective role in fracture cases with poor surrounding soft tissues due to the capability of fracture stabilization with minimal soft tissue disruption and early weight-bearing [8–10]. The hexapod external fixator (HEF), which was initially developed to address multiplanar deformities, consisting of two rings or partial rings connected by six telescopic struts at special universal joints, has become an attractive option in the management of high-energy fractures [5, 11–15]. The HEF simplifies the intraoperative reduction procedures and shortens the operation duration, allowing the treating surgeons to achieve postoperative accurate fracture reduction without frame modification. Compared with the internal fixation techniques, the HEF is associated with lower rates of soft-tissue complications and infection, while comparable bone union rates [16]. The monolateral external fixators (MEF) equipped with the characteristic of easy installation, which seem to be minor discomfort in wearing than circular fixators, is another alternative option for high-energy fractures care but have a limited ability of multiplanar deformities management.

Although both HEF and MEF have been used in lower extremity trauma [4, 12, 14, 15, 17, 18], there are rarely direct comparative studies and the superiority remains uncertain. The purpose of this study was, therefore, to determine the differences of clinical outcomes, if any, between hexapod external fixator and monolateral external fixator in the definitive treatment of high-energy tibial diaphyseal fractures.

## Methods

A total of 53 patients (28 in right tibia, 25 in left tibia) with high-energy tibial diaphyseal fractures and definitively treated by the hexapod external fixator (Tianjin Xinzhong Medical Instrument Co., Ltd., Tianjin, China) or monolateral external fixator (Orthofix, Verona, Italy) were retrospectively collected and analyzed from March 2015 to June 2019, including 43 males and 10 females with an average age of 39 years (range 19 to 64 years). There were 31 patients in the HEF treatment, and the other 22 patients were managed by the MEF.

Inclusion criteria were open fractures, closed fractures with poor surrounding soft tissues where internal fixation was not suitable, or polytrauma with an ISS (Injury Severity Score) ≥16. Patients with neurovascular injury, pathological fractures, poor compliance, age older than 65 years, any other illness that can affect bone healing (such as diabetes), and patients requiring acute lower limb amputation were excluded. Furthermore, patients treated initially with the external fixation but converted to internal fixation were also ineligible. All patients gave written informed consent for their data to be published in our study. This study received approval from the Ethical Committee of our institution.

The open fractures were subdivided depending on the Gustilo and Anderson classification [19]. The demographic data, surgical duration, external fixation time, final radiological results, complications, and clinical outcomes were documented and analyzed. All patients were followed up at a minimum of 12 months after the fixator removal, and none was lost. Any residual deformities in the sagittal or coronal plane were measured using the last available anteroposterior and lateral radiograph of each patient. There was no measurement of limb length discrepancy because the long leg standing radiograph was not routinely done. The clinical outcomes were evaluated by the Association for the Study and Application of the Method of Ilizarov criteria (ASAMI) [20] at the last clinical visit. Difficulties that occurred during the treatment were classified according to Paley [21].

### Surgical technique

The same treating team performed all the surgical procedures, and preventative cephalosporin antibiotics were perioperatively conducted. The patients were positioned supine on a radiolucent table under continuous general or regional anesthesia. For open fractures, radical debridement and sufficient irrigation were performed firstly.

The two kinds of external fixators were applied under image intensifier control to ensure the accuracy of pin insertion. All HEF treatments followed the “ring-first” technique. The two fixator rings were perpendicular to the long axis of the corresponding bony segment in an orthogonal manner. Each bony fragment was fixated by two or three transverse 1.8-mm smooth transosseous wires or one or two 6-mm half pins, and the two rings were independently mounted to these wires and pins on each side [4]. Whenever possible, we prefer to use struts equipped with a fast closure mechanism as they conveniently lock fractures in the desired reduction. The six telescopic struts were attached to the rings and unlocked until the fracture was reduced manually to a grossly acceptable position.

Length and force axis restoration of the injured extremity was achieved firstly when the MEF treatment was conducted. With the temporarily effective fixation of Kirschner wires, three Schanz screws fixed by the connecting rail were inserted on the proximal and distal bony ends, respectively. Every screw needed to be on the same plane. The Kirschner wires were removed subsequently, making sure the fracture was in a stable fixation.

### Postoperative management

For the HEF treatment, the residual deformities were measured by postoperative anteroposterior (AP) and lateral radiographs. The total residual program of the HEF system was performed, and any residual deformities were corrected by gradual strut adjustment postoperatively within 3 days. During the correction procedures, the pain was managed by oral analgesics.

Early active rehabilitation training and progressive staged weight-bearing were the principles of postoperative management. On the postoperative second day, isometric muscle exercise was recommended in all patients. Early partial weight-bearing (supplemented with crutches) was also suggested. The ankle-equines contracture was prevented by a rigid shoe equipped with an elastic band, in which the foot of the injured limb stays in a neutral position. Meticulous pin site care using medical alcohol was conducted every day.

Regular clinical visit and radiographs were conducted monthly for all patients. Callus in three cortices on the AP and lateral radiological images combing with the absence of pain at the fracture site were regarded as the evidence of bone healing. External fixators were dynamized followed by removal. A functional brace was used for refracture prevention for about 1 month.

### Statistical analysis

The SPSS 22.0 (IBM Corp, USA) was used for statistical analysis. Continuous variables were analyzed by Independent-samples T-tests and expressed as the mean, standard deviation (SD), or range of the observations. Count variables were analyzed by the Chi-square or Fisher’s test, representing as a number. A statistically significant difference was set at *P* < 0.05.

## Results

The injury mechanism included road traffic accident in 33 cases, fall from height in 9 cases, crushing injury in 6 cases, and sports injury in 5 cases. There were 37 open fractures and 16 closed fractures. The 37 open fractures consisted of 5 Type I cases, 16 Type II cases, 13 Type IIIA cases, and 3 Type IIIB cases. As for the 16 closed fractures, 2 patients suffered compartment syndrome,11 cases showed severe preoperative hemorrhagic fracture blisters, and the other 3 cases failed closed reduction with plaster immobilization. Eighteen patients had significant associated injuries, including ipsilateral fractures in 8 cases, contralateral fractures in 5 cases, and other fractures in 5 cases. The mean time elapsed since the injury to definitive treatment was 3.3 days (range 1 to 6 days). There were no statistically significant differences in demographics between the two groups (*P* > 0.05) (Table 1).

**Table 1:** Demographics of the two groups

Item	HEF	MEF	*P* value
Patients	31	22	
Gender			
Male	24	19	0.494
Female	7	3	
Age (year)	38.7 ± 10.1	39.6 ± 11.5	0.761
Injury mechanism			
Road traffic accident	20	13	0.810
Fall from height	5	4	
Crushing injury	4	2	
Sports injury	2	3	
Injured bone			
Left tibia	12	13	0.143
Right tibia	19	9	
Open/closed fracture			
Open	22	15	0.828
Closed	9	7	
Gustilo’s classification			
Type I	3	2	0.661
Type II	11	5	
Type IIIA	6	7	
Type IIIB	2	1	
Associated injury			
Ipsilateral fractures	6	2	0.781
Contralateral fractures	2	3	
Other fractures	3	2	
Time elapsed since the injury to definitive treatment (day)	3.2 ± 1.5	3.5 ± 1.7	0.536

*HEF* hexapod external fixator, *MEF* monolateral external fixator

The open wounds were managed by direct closure (5 Type I cases and 14 Type II cases), split-thickness skin grafting (2 Type II cases and 12 Type IIIA cases), and a rotational flap (one Type IIIA case and 3 Type IIIB cases). The compartment syndrome was successfully resolved by fasciotomy followed by delayed skin grafting. The associated fractures in 18 patients were managed simultaneously (8 cases) or subsequently (10 cases).

**Fig. 1:**
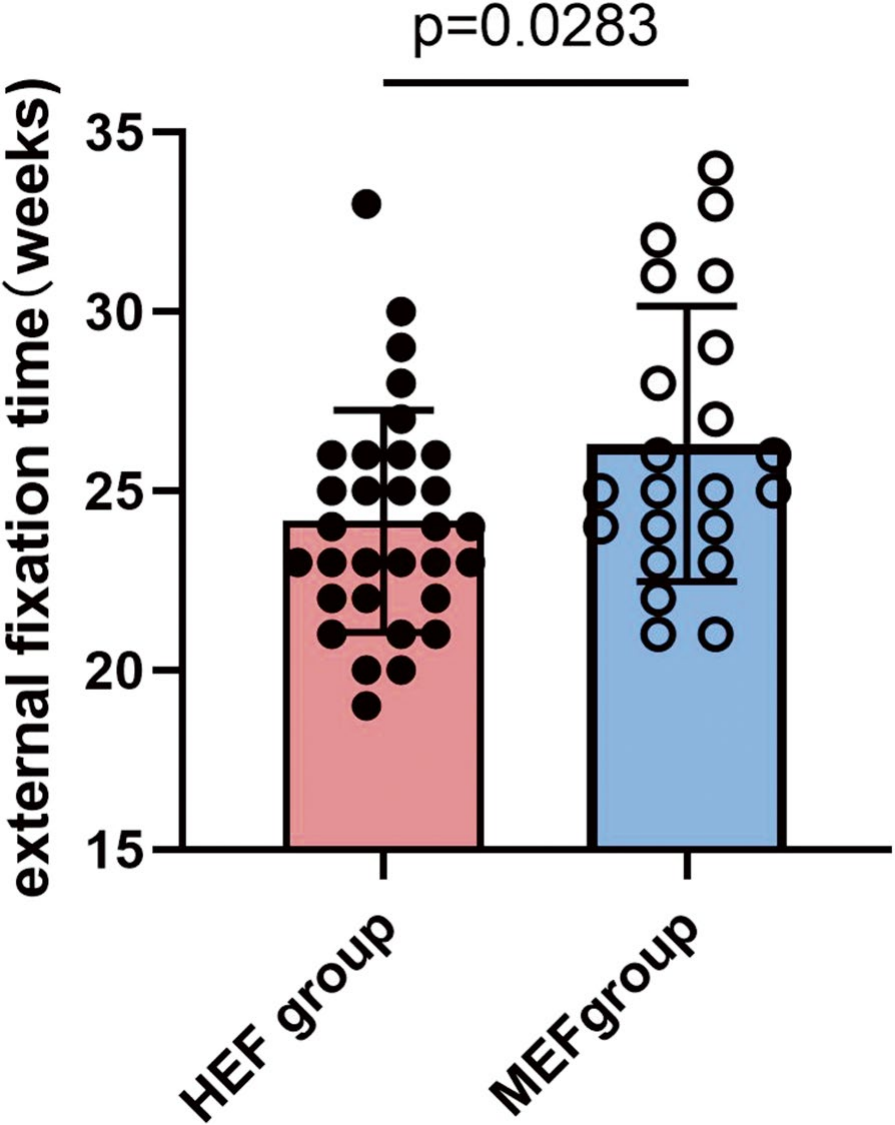
External fixation time between the two groups. Patients in the HEF group (24.2 ± 3.1 weeks, range 19 to 33 weeks) underwent a shorter average external fixation time than that in MEF group (26.3 ± 3.8 weeks, range 21 to 34 weeks) (*P* < 0.05)

The mean surgical duration in the HEF group (62.4 ± 8.3 min) was shorter than that in the MEF group (91.4 ± 6.9 min) (*P* < 0.05). All patients acquired complete bone union finally. Patients in the HEF group (24.2 ± 3.1 weeks, range 19 to 33 weeks) underwent a shorter average external fixation time than that in the MEF group (26.3 ± 3.8 weeks, range 21 to 34 weeks) (*P* < 0.05) (Fig. 1). All patients in both groups were regularly followed up at least 12 months after frame removal, and none was lost (*P* > 0.05) (Table 2).

**Table 2:** Clinical outcomes of the two groups

Item	HEF	MEF	*P* value
Surgical duration (Min)	62.4 ± 8.3	91.4 ± 6.9	*P* < 0.001
External fixation time (week)	24.2 ± 3.1	26.3 ± 3.8	0.028
Follow-up (month)	17.1 ± 4.7	18.4 ± 3.7	0.269
Radiological results			
T1(mm)	1.1 ± 1.0	1.5 ± 1.0	0.136
T2(mm)	0.8 ± 1.1	1.9 ± 1.1	0.001
A1(°)	0.8 ± 0.7	1.1 ± 0.8	0.145
A2(°)	0.5 ± 0.8	1.7 ± 0.9	*P* < 0.001

*HEF* hexapod external fixator, *MEF* monolateral external fixator

T1: Residual translation in the coronal plane

T2: Residual translation in the sagittal plane

A1: Residual angulation in the coronal plane

A2: Residual angulation in the sagittal plane

On the last available radiographs, satisfactory alignment was achieved in all patients without the need for remanipulation. The mean residual translation and angulation in the coronal plane were 1.1 ± 1.0 mm (range 0 to 3 mm) and 0.8 ± 0.7° (range 0 to 2°) for the HEF group, while 1.5 ± 1.0 mm (range 0 to 3.5 mm) and 1.1 ± 0.8° (range 0 to 2°) for the MEF group. There were no statistically significant differences between the two groups regarding the residual deformities in the coronal plane (*P* > 0.05). As for the sagittal plane, the mean residual translation and angulation were 0.8 ± 1.1 mm (range 0 to 3 mm) and 0.5 ± 0.8° (range 0 to 2°) in the HEF group, while 1.9 ± 1.1 mm (range 0 to 4 mm) and 1.7 ± 0.9° (range 0 to 3°) in the MEF group. The residual sagittal plane deformities in the HEF group were all less than that in the MEF group (*P* < 0.05) (Table 2).

No intraoperative complications were observed in the present study. Fifteen patients in the HEF group underwent oral analgesics care during the gradually postoperative reduction, and there was no need for higher-level pain management. Superficial pin tract infection was observed in 13 patients (41.9%) for the HEF group and 6 patients (27.3%) for the MEF group. These patients were successfully treated by daily pin site care and oral antibiotics, and none developed to deep infection requiring surgical intervention. One patient (Type IIIB) in the MEF group suffered postoperative osteomyelitis. The infected and devitalized bone was radically resected, and limb reconstruction using the bone transport technique acquired satisfactory clinical results. Four cases in the MEF group lost the reduction within 2 weeks after the operation and underwent immediate fixator modification in the surgery room. Three patients in the HEF group and 2 patients in the MEF group suffered delayed union, and the bone union was finally achieved by the “accordion maneuver” technique. Nonunion occurred in one case (MEF group) and was treated by autogenous iliac crest bone grafting. Joint stiffness was presented in 2 patients for the HEF group and 2 patients for the MEF group, and resolved by a surgical release along with intensive physiotherapy. No patients of the two groups developed refracture after frame removal. The complication rate was 35.5% in the HEF group, while 45.5% in the MEF group (Table 3).

**Table 3:** Complications of the two groups

Item	HEF (percentage)	MEF (percentage)
Pin tract infection	13(41.9%)	6(27.3%)
Osteomyelitis	0(0%)	1(4.5%)
Loss of reduction	0(0%)	4(18.2%)
Delayed union	3(9.7%)	2(9.1%)
Nonunion	0(0%)	1(4.5%)
Joint stiffness	2(6.5%)	2(9.1%)
Total	18	16
Total patients affected	11	10
Complication rate	35.5%	45.5%

*HEF* hexapod external fixator, *MEF* monolateral external fixator

At the last clinical visit, all the patients have no significant difficulties in their daily activities. According to the ASAMI bone results, there were excellent in 25 patients, good in 5 patients, and fair in 1 patient in the HEF group. As for the MEF group, there were excellent in 16 patients, good in 3, fair in 2, and poor in 1. For the ASAMI functional results, in the HEF group, there were excellent in 16 patients, good in 12, and fair in 3. In the MEF group, there were excellent in 13 patients, good in 7, and fair in 2. There was no statistically significant difference between the two groups in ASAMI scores (*P* > 0.05) (Table 4).

**Table 4:** Results of ASAMI scores in the two groups

Item	Excellent	Good	Fair	Poor	Failure	*P* value
Bone results						
HEF	25	5	1	0	–	0.503
MEF	16	3	2	1	–	
Functional results						
HEF	16	12	3	0	0	0.858
MEF	13	7	2	0	0	

ASAMI Criteria:

Bone results

Excellent: Union, no infection, deformity < 7°, limb length discrepancy (LLD) < 2.5 cm

Good: Union plus any two of the following: absence of infection, deformity < 7°, LLD < 2.5 cm

Fair: Union plus any one of the following: absence of infection, deformity < 7°, LLD < 2.5 cm

Poor: Nonunion/refracture/union plus infection plus deformity > 7° plus LLD > 2.5 cm

Functional results

Excellent: Active, no limp, minimum stiffness (loss of < 15°knee extension/< 15°ankle dorsiflexion) no reflex sympathetic dystrophy (RSD), insignificant pain

Good: Active, with one or two of the following: limb, stiffness, RSD, significant pain

Fair: Active, with three or all of the following: limb, stiffness, RSD, significant pain

Poor: Inactive (unemployment or inability to return to daily activities because of injury)

Failure: Amputation

*HEF* hexapod external fixator, *MEF* monolateral external fixator

**Fig. 2:**
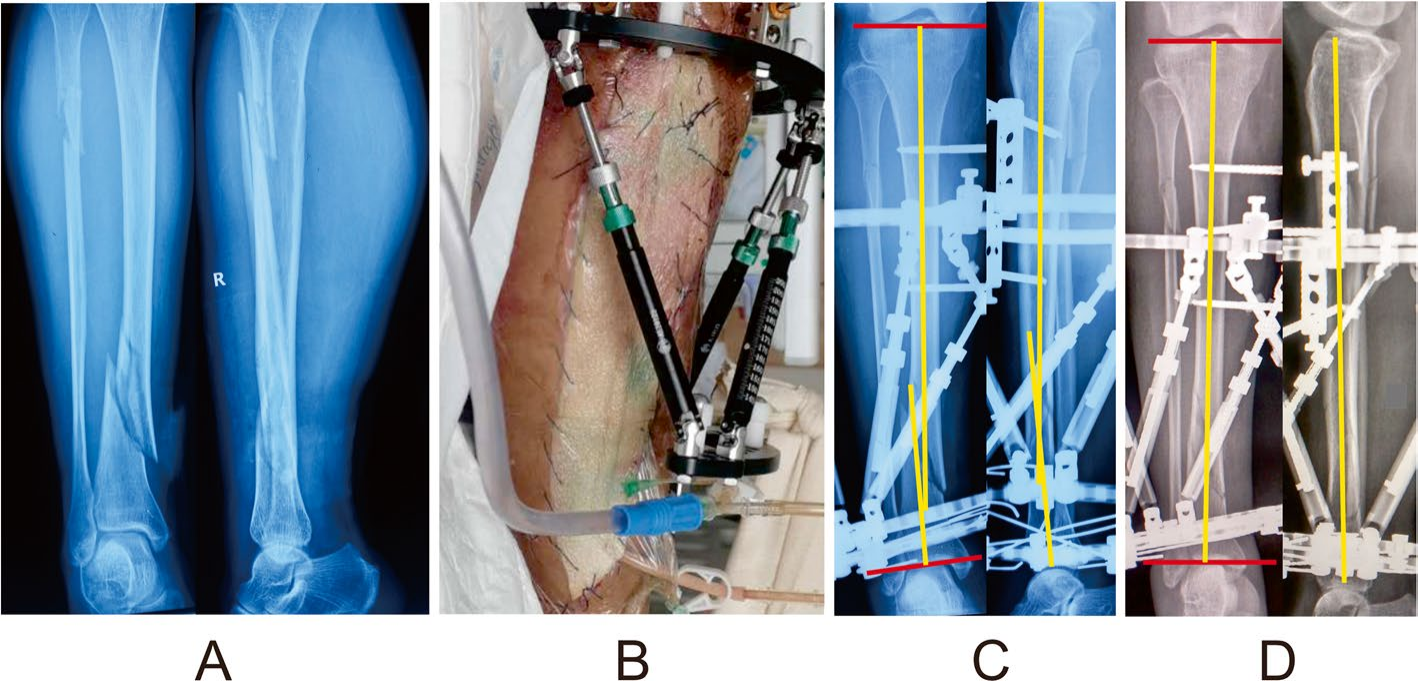
Images of a 39-year-old man with multidimensional deformities in tibia and fibula caused by a road traffic accident and treated by the HEF. **a** Posttraumatic radiographs. **b** Patient with compartment syndrome resolved by fasciotomy combing with vacuum sealing drainage technique. **c** Radiographs immediately after the operation, manifesting varus and flexion residual deformities that needed to be corrected. **d** Radiographs after final correction, showing satisfactory alignment

**Fig. 3:**
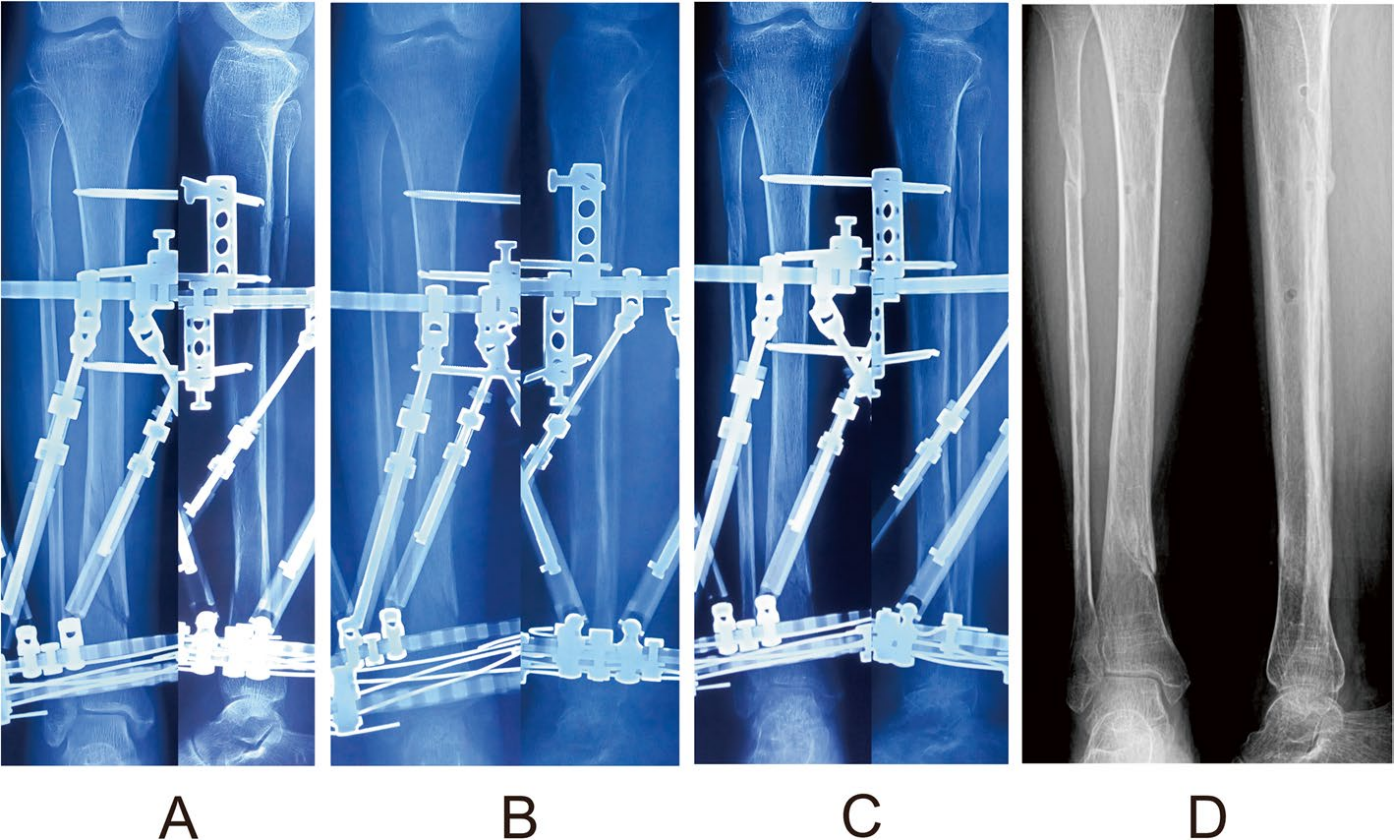
Follow-up radiographs of the same patient after final correction. **a** Radiographs 1 month later. **b** Radiographs 3 months later. **c** Radiographs 5 months later. **d** Radiographs 6 months later after the frame removal

A typical case in the HEF treatment is shown in Figs. 2 and 3.

## Discussion

The tibial diaphyseal fractures are usually caused by high-energy trauma [4, 11, 22], and the relatively superficial location makes the tibia more susceptible to open fractures associated with significant soft tissue damage and bone loss, resulting in nonunion and deep infection [16]. Optimal management remains a controversial problem. Previous studies have emphasized the stable fixation and minimal soft tissue disruption for these complex fractures, thereby maintaining the biomechanical microenvironment which is beneficial to bone healing [10, 23–25]. Preventing infection, obtaining union, and returning the normal daily life is the ultimate goal.

Although intramedullary nail is the gold standard in the management of tibial diaphyseal fractures, external fixation has a clear role in most cases due to the advantage of stabilization with limiting soft tissue dissection, especially for high-energy injuries with poor surrounding soft tissues [3, 4, 6, 10, 11, 15, 18, 22, 26]. Intramedullary nails work best in managing diaphyseal transverse fractures, but there are challenges in unstable oblique, spiral, and comminuted fractures [27]. Fortunately, kinds of external fixators provide a stable frame for the varying patterns of tibial fractures. Liu et al. [4] conducted hexapod external fixation treatment in 34 high-energy tibial shaft fractures, and the results manifested that the HEF is an alternative and effective method, including various technical advantages. Mangukiya et al. [18] achieved satisfactory clinical outcomes in the treatment of 40 patients with compound tibia diaphyseal fracture using an AO monolateral external fixator or Limb reconstruction system. Dickson, D R et al. [16] reported on the surgical and functional outcomes of 22 patients with Grade 3 open tibial fractures treated with a circular frame. All cases united, and there were no re-fractures or amputations.

Both circular and monolateral external fixation have been well described in treating tibial shaft fractures with success [4, 15–18, 28]. For trauma-control and definitive management, the monolateral external fixators are more likely to be accepted by patients due to wearing-convenient, as well as the treating surgeons because of more accessible application with fewer parts and modifications. Still, they are limited in deformity correction due to the inherent characteristic of uniplanar fixation. The circular external fixators tend to be discommodious to patients, but are more versatile in treatment procedures. Although the superior circular fixator patterns remain uncertain, the HEF, which allows immediate trauma-control and accurate fracture reduction without frame alteration, has become an attractive option as more general orthopedic surgeons are familiar with this device in recent years.

The current study reported a group of 53 high-energy tibial diaphyseal fractures treated by HEF or MEF. Several complications in external fixation treatment, such as pin tract infection, loss of reduction, delayed union, nonunion, and joint stiffness, have been well reported [4, 11, 29]. In reviewing our data, pin tract infection was the most common complication, as expected. The total pin tract infection rate was 35.8%, matching the previous literature of Francesco et al. [30] (35%) and Antoci et al. (33%) [31]. The differences between the HEF group (41.9%) and the MEF group (27.3%) may be explained due to the more wires and half pins in the HEF group.

We also noted a high reduction loss rate leading to return to the operating room for remanipulation in the MEF group (18.2%), but none was observed in the HEF group. Furthermore, reduction loss was commonly occurred in cases with a relatively small contact area and had relatively little inherent stability. With the substantial difference in design between the HEF and MEF, we do not think that the MEF should not be used to manage tibial diaphyseal fractures, but rather, a circular fixation should be considered in unstable fracture patterns. Alternatively, if a MEF is used for oblique or comminuted fractures, the fracture alignment should be particularly concerned by the treating surgeon.

Although there was a similar delayed union rate between the HEF group (9.7%) and the MEF group (9.1%), the external fixation time in the HEF group (24.2 ± 3.1 weeks) was shorter than that in the MEF group (26.3 ± 3.8 weeks). Additionally, the joint stiffness rate in the HEF group (6.5%) was lower than that in the MEF group (9.1%). The fewer external fixation duration in the HEF group may explain this problem. Furthermore, in the MEF group, nonunion was observed in one case and successfully treated by autogenous iliac crest bone grafting. Compared with uniplanar fixation in MEF treatment, we speculate that the HEF with multiplanar fixation provides a more stable mechanical microenvironment which is beneficial to fracture healing. Another patient in the MEF group was also observed to suffer osteomyelitis and resolved by bone transport technique. The complication rate was 35.5% in the HEF group, while 45.5% in the MEF group. Statistically significant differences were not observed in the ASAMI scores as the sample size was insufficient to reach adequate power, but the observed clear trend implies that there was shorter surgical duration and external fixation time in the HEF treatment, as well as fewer complications.

The hexapod external fixator provides the ability to achieve excellent alignment postoperatively, resulting in a rapid installation and less duration in the operating room even in inexperienced hands without worrying about the accuracy of fracture reduction. Although all the 53 patients in this study achieved functional reduction, there was statistical significance in the residual deformities on the sagittal plane between the two groups. This may be explained that the treating surgeons may subjectively overlook deformities in the sagittal plane during the surgical procedures, while these residual deformities can be salvaged by the postoperative adjustment using a hexapod external fixator. The other possible explanation for these differences could be the relatively unstable eccentric fixation in the MEF group resulting in some deformity recurrence.

Our study preliminary compared the clinical outcomes between hexapod external fixator and monolateral external fixator in the definitive treatment of high-energy tibial diaphyseal fractures. The results manifested that the hexapod external fixation is a superior effective treatment for the high-energy tibial diaphyseal fractures with the advantages of stable fixation, less surgical duration, postoperatively satisfactory fracture reduction, and fewer complications. However, the high cost and long learning curve, we think, may be the significant limitations of the hexapod external fixation.

The present study has several limitations. Firstly, the selection bias may derive from the retrospective nature. In addition, a conservative attitude should be adopted regarding the interpretations of our results due to a single-center small sample size. A further study with a multi-center large sample size is needed. Furthermore, statistically significant differences based on smaller differences are most likely clinically not relevant and therefore meaningless. Despite these limitations, this study directly compares the clinical outcomes between the HEF and MEF in the definitive treatment of high-energy tibial diaphyseal fractures and preliminarily draws a conclusion.

## Conclusion

There is no statistically significant difference in finally clinical outcomes between hexapod external fixator and monolateral external fixator in the definitive treatment of high-energy tibial diaphyseal fractures. The hexapod external fixation treatment is a superior effective method, including advantages of stable fixation, less surgical duration, postoperatively satisfactory fracture reduction, and fewer complications.

## Data Availability

The datasets analyzed during the current study are available from the corresponding author on reasonable request.

## Abbreviations

HEF: Hexapod external fixator
MEF: Monolateral external fixator
ASAMI: The Association for the Study and Application of the Method of Ilizarov
ORIF: Open reduction and internal fixation
AP: Anteroposterior
ROM: Range of motion

## References

[1] Rittstieg P, Wurm M, Muller M, Biberthaler P (2020). Current treatment strategies for lower leg fractures in adults. Unfallchirurg.

[2] French B, Tornetta PR (2002). High-energy tibial shaft fractures. Orthop Clin North Am.

[3] Golubovic I, Vukasinovic Z, Stojiljkovic P, Golubovic Z, Stamenic S, Najman S (2012). Open segmental fractures of the tibia treated by external fixation. Srp Arh Celok Lek.

[4] Liu Y, Liu J, Yushan M, Liu Z, Zhang T, Ma H (2021). Management of high-energy tibial shaft fractures using the hexapod circular external fixator. BMC Surg.

[5] Henderson DJ, Barron E, Hadland Y, Sharma HK (2015). Functional outcomes after tibial shaft fractures treated using the Taylor spatial frame. J Orthop Trauma.

[6] Rogers GP, Tan HB, Foster P, Harwood P (2019). Complex Tibial shaft fractures in children involving the distal Physis managed with the Ilizarov method. Strategies Trauma Limb Reconstr.

[7] Wani N, Baba A, Kangoo K, Mir M (2011). Role of early Ilizarov ring fixator in the definitive management of type II, IIIA and IIIB open tibial shaft fractures. Int Orthop.

[8] Grubor P, Milicevic S, Grubor M, Meccariello L (2015). Treatment of bone defects in war wounds: retrospective study. Med Arch.

[9] Rollo G, Falzarano G, Ronga M, Bisaccia M, Grubor P, Erasmo R (2019). Challenges in the management of floating knee injuries: results of treatment and outcomes of 224 consecutive cases in 10 years. Injury.

[10] Has B, Jovanovic S, Wertheimer B, Mikolasevic I, Grdic P (1995). External fixation as a primary and definitive treatment of open limb fractures. Injury.

[11] Potgieter MS, Pretorius HS, Preez GD, Burger M, Ferreira N (2020). Complications associated with hexapod circular fixation for acute fractures of the tibia diaphysis: a retrospective descriptive study at a high volume trauma Centre. Injury.

[12] Iobst CA (2016). Hexapod external fixation of tibia fractures in children. J Pediatr Orthop.

[13] Wei M, Chen J, Guo Y, Sun H (2017). The computer-aided parallel external fixator for complex lower limb deformity correction. Int J Comput Assist Radiol Surg.

[14] Sala F, Elbatrawy Y, Thabet AM, Zayed M, Capitani D (2013). Taylor spatial frame fixation in patients with multiple traumatic injuries: study of 57 long-bone fractures. J Orthop Trauma.

[15] Al-Sayyad MJ (2008). Taylor spatial frame in the treatment of open tibial shaft fractures. Indian J Orthop.

[16] Dickson DR, Moulder E, Hadland Y, Giannoudis PV, Sharma HK (2015). Grade 3 open tibial shaft fractures treated with a circular frame, functional outcome and systematic review of literature. Injury.

[17] Messner J, Harwood P, Johnson L, Itte V, Bourke G, Foster P (2020). Lower limb paediatric trauma with bone and soft tissue loss: Ortho-plastic management and outcome in a major trauma Centre. Injury.

[18] Mangukiya HJ, Mahajan NP, Pawar ED, Mane A, Manna J (2018). Functional and radiological outcome in management of compound tibia diaphyseal fracture with AO monolateral fixator versus limb reconstruction system. J Orthop.

[19] Gustilo RB, Anderson JT (1976). Prevention of infection in the treatment of one thousand and twenty-five open fractures of long bones: retrospective and prospective analyses. J Bone Joint Surg Am.

[20] Paley D, Catagni MA, Argnani F, Villa A, Benedetti GB, Cattaneo R. Ilizarov treatment of tibial nonunions with bone loss. Clin Orthop Relat Res. 1989:146–65. 2924458

[21] Paley D. Problems, obstacles, and complications of limb lengthening by the Ilizarov technique. Clin Orthop Relat Res. 1990:81–104. 2403498

[22] Alhammoud A, Maaz B, Alhaneedi GA, Alnouri M (2019). External fixation for primary and definitive management of open long bone fractures: the Syrian war experience. Int Orthop.

[23] Giotakis N, Panchani SK, Narayan B, Larkin JJ, Al MS, Nayagam S (2010). Segmental fractures of the tibia treated by circular external fixation. J Bone Joint Surg Br.

[24] Sarmiento A, Latta LL (2008). Functional treatment of closed segmental fractures of the tibia. Acta Chir Orthop Traumatol Cechoslov.

[25] Fowler T, Whitehouse M, Riddick A, Khan U, Kelly M (2019). A retrospective comparative cohort study comparing temporary internal fixation to external fixation at the first stage debridement in the treatment of type IIIB open Diaphyseal Tibial fractures. J Orthop Trauma.

[26] Falzarano G, Pica G, Medici A, Rollo G, Bisaccia M, Cioffi R (2018). Foot loading and gait analysis evaluation of nonarticular Tibial Pilon fracture: a comparison of three surgical techniques. J Foot Ankle Surg.

[27] Ricci WM, O'Boyle M, Borrelli J, Bellabarba C, Sanders R (2001). Fractures of the proximal third of the tibial shaft treated with intramedullary nails and blocking screws. J Orthop Trauma.

[28] Bartlett CR, Weiner LS, Yang EC (1997). Treatment of type II and type III open tibia fractures in children. J Orthop Trauma.

[29] Liu Y, Yushan M, Liu Z, Liu J, Ma C, Yusufu A (2020). Complications of bone transport technique using the Ilizarov method in the lower extremity: a retrospective analysis of 282 consecutive cases over 10 years. BMC Musculoskelet Disord.

[30] Sala F, Thabet AM, Capitani P, Bove F, Abdelgawad AA, Lovisetti G (2017). Open supracondylar-Intercondylar fractures of the femur treatment with Taylor spatial frame. J Orthop Trauma.

[31] Antoci V, Ono CM, Antoci VJ, Raney EM (2008). Pin-tract infection during limb lengthening using external fixation. Am J Orthop (Belle Mead NJ).

